# Towards quantitative ionizing radiation acoustic imaging (iRAI) for radiation dose measurement: Validation from simulations to experiments

**DOI:** 10.1002/mp.18091

**Published:** 2025-09-01

**Authors:** Yaocai Huang, Lise Wei, Dale Litzenberg, Borui Li, Chenshuo Ma, Hyeonwoo Kim, Yiming Liu, Claire Zhang, Paul L. Carson, Issam El Naqa, Wei Zhang, Xueding Wang

**Affiliations:** ^1^ Department of Biomedical Engineering University of Michigan Ann Arbor Michigan USA; ^2^ Department of Radiation Oncology University of Michigan Ann Arbor Michigan USA; ^3^ Department of Electrical and Computer Engineering University of Michigan Ann Arbor Michigan USA; ^4^ Department of Radiology University of Michigan Ann Arbor Michigan USA; ^5^ Department of Machine Learning Moffitt Cancer Center Tampa Florida USA; ^6^ Department of Radiation Oncology Moffitt Cancer Center Tampa Florida USA

**Keywords:** dose mapping, ionizing radiation acoustic imaging, simulation and experimental validation, transducer detection sensitivity, treatment plan

## Abstract

**Background:**

In clinical radiation therapy (RT), accurately quantifying the delivered radiation dose to the targeted tumors and surrounding tissues is essential for evaluating treatment outcomes. Ionizing radiation acoustic imaging (iRAI), a novel passive and non‐invasive imaging technique, has the potential to provide real‐time in vivo radiation dose mapping during RT. However, current iRAI technology does not account for spatial variations in the detection sensitivity of the ultrasound transducer used to capture the iRAI signals, leading to significant errors in dose mapping.

**Purpose:**

This paper presents the first detection sensitivity‐compensated quantitative iRAI approach for measuring deposited radiation dose, aiming at improving dose mapping accuracy.

**Methods:**

Detection sensitivity maps for the 2D matrix array transducer (MAT) were generated through both computational studies and experimental measurements. First, the Field II MATLAB toolbox was used to simulate the acoustic fields generated by the 2D MAT at various focal angles in the region of interest. Second, the prototype 2D MAT was applied to experimentally measure the acoustic signals generated by pulsed laser point sources distributed throughout the same volume as in the simulation. Then, in vitro experiments were conducted using homogeneous soft‐tissue phantoms, where x‐ray beams with square fields and a C‐shaped treatment plan were separately delivered via a clinical linear accelerator (LINAC). Additionally, the propagation of acoustic waves induced by the x‐ray beams with square fields was simulated using the K‐Wave MATLAB toolbox. Correction factors derived from both the simulated and experimental sensitivity maps were applied to compensate for sensitivity‐induced discrepancies in the iRAI reconstruction results. Dose distributions in uncompensated and sensitivity‐compensated iRAI volumetric images were compared across various beam positions and field sizes. The agreement between the iRAI images and the treatment plan was quantitatively evaluated using structural similarity index measure (SSIM) and gamma index analysis.

**Results:**

The experimental results, including the detection sensitivity map and iRAI measurements of x‐ray beams with square fields, showed strong agreement with the corresponding simulated outcomes. Following compensation, the relative amplitudes of all iRAI images for beams targeting different positions converged toward 1. The compensated iRAI images revealed greater agreement with the treatment plan in dose distribution, compared to the pre‐compensation images. This improvement was further supported by global gamma index analysis, which showed an increase in the 5%/5 mm dose difference (DD) /distance‐to‐agreement (DTA) passing rate from 56.86% to 78.24% after compensation, indicating improved accuracy in reconstructing the dose distribution.

**Conclusions:**

This study demonstrated that addressing inhomogeneities in transducer detection sensitivity significantly enhances the accuracy of radiation dose mapping by iRAI.

## 1 INTRODUCTION

Radiation therapy (RT), which uses high‐energy ionizing radiation to target malignant cells and damage their DNA, is widely applied in cancer treatment.^1^, ^2^ The effectiveness of RT depends critically on delivering precise radiation doses to tumors while minimizing exposure to surrounding healthy tissues.^3^, ^4^ Advancements in dose delivery technologies such as intensity‐modulated radiation therapy (IMRT) and volumetric modulated arc therapy (VMAT) have significantly improved the precision and efficacy of cancer treatments by optimizing radiation delivery strategies and reducing treatment times.^5^, ^6^, ^7^ Advanced imaging technologies, such as computed tomography (CT), magnetic resonance imaging (MRI), and positron emission tomography (PET), have been integrated into radiation therapy to enhance tumor localization during treatment sessions, forming the basis of image‐guided radiation therapy (IGRT).^8^, ^9^ Although IGRT improves targeting precision and minimizes radiation exposure to nearby healthy tissues, it cannot visualize actual dose deposition within anatomical structures and therefore cannot provide real‐time feedback on potential dose‐tumor misalignment.^10^ This highlights the need for new technologies that can accurately monitor dose delivery deep into the body in real time.

One promising solution to this challenge is ionizing radiation acoustic imaging (iRAI), a novel technique that can visualize radiation dose deposition in real time by detecting acoustic waves induced by the rapid temperature rise from radiation energy absorption in tissues.^11^ Unlike traditional dose measurement tools such as diodes and films,^12^ and clinical in vivo dosimeters including thermoluminescent dosimeters^13^ and optically stimulated luminescent dosimeters,^14^ which rely on heat‐ or light‐induced signals from irradiated materials, iRAI offers real‐time, volumetric imaging that directly correlates with the deposited radiation dose in tissue, provided that factors such as tissue density, acoustic attenuation, speed of sound, and thermal expansion are properly accounted for. This provides a more comprehensive and dynamic view of dose distribution in RT clinical settings. Previous studies have demonstrated the feasibility of iRAI in simulations, phantom experiments, and animal models, marking significant progress toward clinical translation.^11^, ^15^, ^16^, ^17^, ^18^, ^19^, ^20^, ^21^ For instance, iRAI has been successfully integrated with ultrasound to enable dual‐modality imaging in rabbit models, facilitating simultaneous visualization of dose deposition and real‐time tracking of tissue movement.^22^, ^23^ More recently, we developed a clinical‐grade iRAI system utilizing a 2D matrix array transducer (MAT), and, for the first time, achieved real‐time radiation dose mapping in a patient's liver during RT.^10^ With a unique capability to provide real‐time, volumetric dose information in vivo, iRAI holds great potential for adaptive RT (ART) in clinical settings.

While iRAI has demonstrated potential for visualizing radiation dose distribution, quantitative dose mapping remains an unresolved challenge in current implementations. One key limitation arises from spatial variations in transducer detection sensitivity, which are not yet compensated for during delay‐and‐sum‐based image reconstruction. These uncorrected variations lead to signal discrepancies when the relative position of the radiation dose with respect to the transducer changes, making it difficult to achieve uniform and accurate dose representations across the imaging volume. In this study, we developed compensation factors to correct spatial variations in transducer sensitivity, enabling more accurate 3D dose mapping. Their effectiveness was evaluated through simulations and further validated experimentally using phantoms with a clinically relevant treatment plan.

## 2 METHODS

### 2.1 Transducer detection sensitivity characterization

**FIGURE 1:**
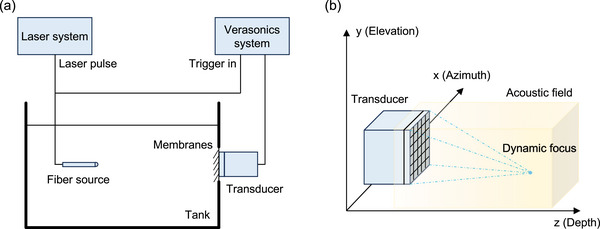
Graphic illustration of detection sensitivity characterization. (a) Block diagram of the 2D MAT detection sensitivity measurement experiment. (b) Schematic of the 2D MAT detection sensitivity simulation using the Field II MATLAB toolbox.

In this study, a 2D MAT, driven by a Vantage System (Verasonics Inc), was used to acquire ionizing radiation acoustic signals. The technical specifications of the 2D MAT, as detailed in our previous publication, are summarized in Table 1.^10^ In brief, a central frequency of 0.35 MHz was selected for the 2D MAT to match the power spectrum of the radiation‐induced acoustic signals generated by the 4 µs‐duration x‐ray pulses. The graphic illustration of the detection sensitivity experiment in a homogeneous medium is shown in Figure 1a. A 532 nm pulsed laser (GLPM‐10, IPG Photonics, Inc.) with a 4 ns pulse duration and a 500 Hz repetition rate was used to generate photoacoustic waves. The laser light was delivered through a 600 µm‐core optical fiber (FT600EMT, Thorlabs, Inc.). The fiber was immersed in a water tank and aligned toward the 2D MAT. The distal end of the fiber was coated with a UV‐curable adhesive (GETLMUL, Inc.) to form a hemispherical tip, which was then coated with a thin gold layer, creating a spherical acoustic source when laser light was delivered through the fiber and hit the gold coating. The 2D MAT was affixed to the exterior of the water tank and acoustically coupled to the water via a thin membrane. During laser emission, synchronized trigger pulses from the laser system initiated data acquisition by the Verasonics system. The fiber was manually moved through a 3D space of 15 cm (azimuth) × 15 cm (elevation) × 25 cm (depth) with a consistent step size of 2.5 cm in each direction. At each location, the laser‐induced acoustic signals were acquired and averaged over 1000 pulses to ensure reliability.

**TABLE 1:** Technical specifications of the 2D matrix array transducer.

Center frequency (MHz)	Bandwidth	Number of elements	Element size (mm)	Kerf (mm)
0.35	50%	32 × 32 = 1024	3.45 × 3.45	0.2

The 2D MAT comprises 1024 transducer elements, while the Vantage system supports only 256 acquisition channels. A 4‐to‐1 multiplexer was therefore used to enable full data acquisition. The data acquired from 1000 laser pulses were equivalent to 250 full acquisitions. Signals were averaged250 times and subsequently reconstructed into a single image frame using a delay‐and‐sum image reconstruction algorithm combined with envelope detection in MATLAB 2024a (MathWorks). This processing ultimately produced a 3D photoacoustic image, representing the spatial distribution of the photoacoustic signal intensities. After reconstructing images from signals collected at various positions inside the water tank, the maximum amplitude from each image was extracted to represent the detection sensitivity of the 2D MAT at that position.

To verify the effectiveness of the detection sensitivity obtained from the experiment, simulations were performed in parallel to generate a simulated detection sensitivity map. These simulations were conducted using the Field II MATLAB toolbox (Version 3.30), based on the actual parameters of the 2D MAT. The computational environment included Windows 10 Education, dual Intel Xeon E5‐2640 v4 processors at 2.40 GHz, 384 GB RAM, and 20 parallel processing units to enhance computational efficiency. A 3D space of 15 × 15 × 25 cm^3^ was established for the simulation. As illustrated in Figure 1b, a dynamic focusing approach was used to sweep through the volumetric space and sequentially capture the acoustic field at different focal angles. The dynamic focusing was achieved by adjusting the angles in both the azimuth (0° to 180°) and elevation (0° to 180°) directions, with a step size of 0.5°, resulting in 129600 (360 × 360) focusing events. For each focusing event, an individual acoustic field was calculated. At each voxel within the measured space, the maximum value across all acoustic fields was selected, ultimately generating the simulated detection sensitivity map.

### 2.2 Radiation beam study in phantom

**FIGURE 2:**
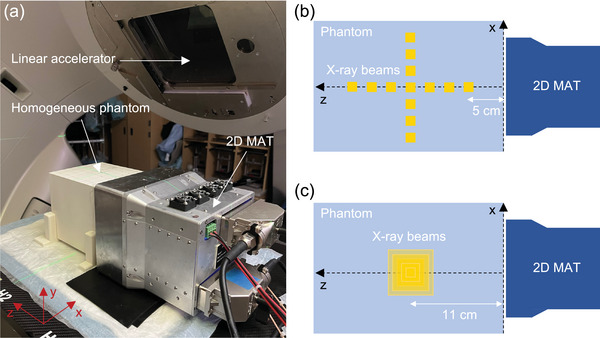
Experimental setup for the radiation beam study. (a) Photograph of the x‐ray beam imaging experiment conducted in the radiation therapy room. (b) Schematic of x‐ray beams delivered every 2 cm along the central axis from the transducer surface into the homogeneous phantom. At a depth of 11 cm, six additional lateral positions were targeted, spaced 2 cm apart. Each beam has a field size of 1 cm × 1 cm. (c) Schematic showing x‐ray beams of varying field sizes (1 × 1 cm², 2 × 2 cm², 3 × 3 cm², 4 × 4 cm², and 5 × 5 cm²) delivered to a fixed depth of 11 cm along the central axis from the transducer surface.

To evaluate the spatial non‐uniformity of iRAI due to detection sensitivity variation, experiments were conducted using a homogeneous soft‐tissue phantom. As shown in Figure 2a, a LINAC (Truebeam, Varian Medical Systems, Inc.) was configured to vertically irradiate a rectangular homogeneous phantom composed of solidified vegetable oil, completely enclosed in a box (17 cm × 17 cm × 20 cm) made of biopolymer. Upon mixing with an oil solidifier (FRYAWAY Inc.), the vegetable oil solidified with a density of 920 kg/m^3^ and a speed of sound of 1445 m/s. The 2D MAT was attached to one side of the phantom and coupled via ultrasound gel to capture radiation‐induced acoustic signals. The LINAC operated at a dose rate of 1400 MU/min (1 MU = 0.8 cGy), with a repetition rate of 330 Hz, delivering a total dose of 2.26 Gy per measurement. Beam delivery plans are illustrated in Figure 2b and 2c. The x‐ray beams, with a size of 1 cm by 1 cm, were vertically directed to the phantom, along the central axis at distances of 5 cm, 7 cm, 9 cm, 11 cm, 13 cm, 15 cm, and 17 cm from the transducer surface. At 11 cm, seven lateral positions distributed within the phantom were targeted by the same beams, each separated by 2 cm. Additionally, x‐ray beams of varying sizes (1 × 1 cm², 2 × 2 cm², 3 × 3 cm², 4 × 4 cm², and 5 × 5 cm²) were delivered to a target depth of 11 cm. Each beam configuration, whether based on position or size, was delivered three times for statistical analysis of the imaging results. Correspondingly, iRAI of the x‐ray beams was simulated using the K‐Wave MATLAB toolbox based on the experimental setup and conditions. The simulation was conducted on a system running Windows 10 Pro, equipped with an Intel Core i7‐10750H processor (2.60 GHz), 16 GB DDR4 RAM, a 512 GB SSD, and utilizing 7 parallel processing units to improve computational efficiency. To obtain a standard dose deposition result, the beams were also measured with radiochromic films (EBT3, Ashland Inc.), a widely accepted method in clinical dosimetry.^24^

### 2.3 Clinical treatment plan study

**FIGURE 3:**
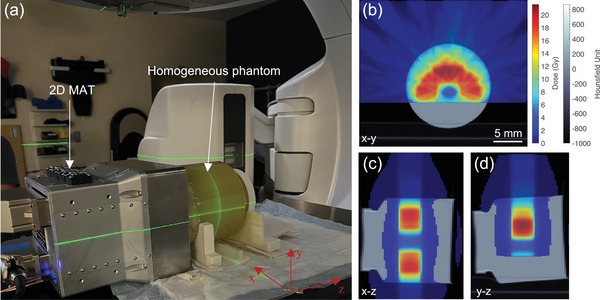
Experimental setup for iRAI validation with a clinical treatment plan. (a) Photograph of the phantom positioning and irradiation setup in the radiation therapy room. (b) Axial (x‐y), (c) Coronal (x‐z), and (d) Sagittal (y‐z) cross‐sectional views of the treatment plan, respectively. In these images, the gray areas represent CT scans, and the heatmaps indicate the planned dose distribution.

In this section, we conducted a phantom study based on a clinically relevant C‐shaped radiotherapy treatment plan, which is commonly designed to target tumors while preserving critical organs such as the spine. A cylindrical homogeneous soft‐tissue phantom was used to simulate the tissue environment. A CT scan of the phantom was performed for treatment planning purposes. The treatment plan was calculated using a clinical treatment planning system (Varian Medical Systems, Inc.), with a maximum dose of 21.6 Gy. An in‐room laser system was used to position both the phantom and the 2D MAT. Axial, sagittal, and coronal cross‐sectional views of the treatment plan, with CT images overlaid, are shown in Figure 3b, 3c, and 3d, respectively. The dark gray areas represent the CT scans, while the heatmap regions illustrate the dose distribution. During radiation delivery, the isocenter was aligned with the geometric center of the phantom using an in‐room laser system, and the 2D MAT was coupled to one side of the phantom with ultrasound gel.

### 2.4 Data processing and quantification

The raw data acquired from both experiments and simulations were processed using a 3D delay‐and‐sum reconstruction algorithm on MATLAB. The correction factors used to compensate for the post‐reconstruction images were the reciprocals of the detection sensitivity maps. Correction factors derived from experimental measurements and simulations were applied to their respective data sets.

The dose volume histogram (DVH) is a commonly used method for summarizing 3D dose‐volume data,^25^ where each point on the histogram represents the relative volume receiving a dose equal to or greater than a specified level. In this study, DVH was utilized to visualize the similarity between dose distributions derived from the experimental and simulated detection sensitivity maps. For the images of beams directed at the homogeneous phantom, their amplitudes were first normalized. Image voxels within the dose distribution were then averaged to obtain a normalized amplitude representing the targeted position.

To assess beams of varying sizes, depth dose profiles were extracted along the midline of the reconstructed images to evaluate and compare dose distributions. To quantitatively assess the effectiveness of the proposed iRAI approach in improving dose mapping accuracy, the C‐shaped treatment plan and corresponding iRAI images were evaluated using isodose lines, structural similarity index measure (SSIM), and Gamma index analysis with 5%/5 mm dose‐difference (DD) / distance‐to‐agreement (DTA).^26^ SSIM assesses image similarity by comparing structural information, luminance, and contrast between two images, providing a quantitative measure of agreement between the planned dose distribution and the acquired images.^27^ Gamma index analysis, widely used in radiotherapy, evaluates the agreement between planned and delivered dose distributions by comparing both the dose values and their spatial locations, ensuring precision and safety, particularly in protecting critical structures such as organs at risk.^28^

## 3 RESULTS

### 3.1 Transducer detection sensitivity and correction factors

**FIGURE 4:**
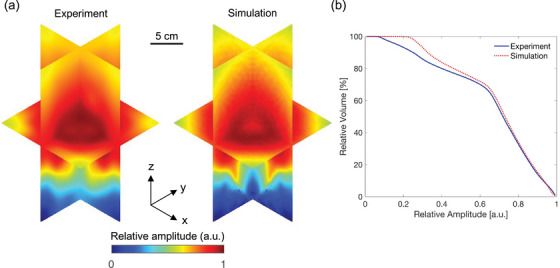
Detection sensitivity characterization of the 2D MAT. (a) Orthogonal cross‐sectional views of simulated and experimental detection sensitivity maps. The color bar indicates relative amplitude in arbitrary units (a.u.), ranging from 0 (blue) to 1 (red). The scale bar represents 5 cm. The orientation of the sensitivity maps follows the indicated coordinate system, where the x‐, y‐, and z‐axes correspond to azimuth, elevation, and depth, respectively. (b) Comparison of DVH curves derived from simulated and experimental volumetric detection sensitivity maps.

The volumetric detection sensitivity maps measured experimentally and generated using the Field II MATLAB simulation are shown in Figure 4a. The experimental detection sensitivity map shows high sensitivity around the transducer's focal zones, approximately 15 cm from the transducer surface, which gradually decreases with distance. The simulated sensitivity map presents a more symmetrical and uniform distribution due to the idealized conditions in the simulation model. Although the experimental map maintains the overall shape and trend observed in the simulation, it exhibits lower amplitudes and less defined boundaries, particularly in the peripheral regions. The DVH curves, presented in Figure 4b, show the relationship between relative amplitude and the percentage of volume for both experimental and simulated detection sensitivity. Both curves follow a similar trajectory, with approximately 80% of the volume achieving a relative amplitude above 0.5 a.u. The correction factors were generated by taking the reciprocal of the detection sensitivity maps, with the experimental and simulated volumes derived from their respective sensitivity maps.

### 3.2 Radiation beam quantification

**FIGURE 5:**
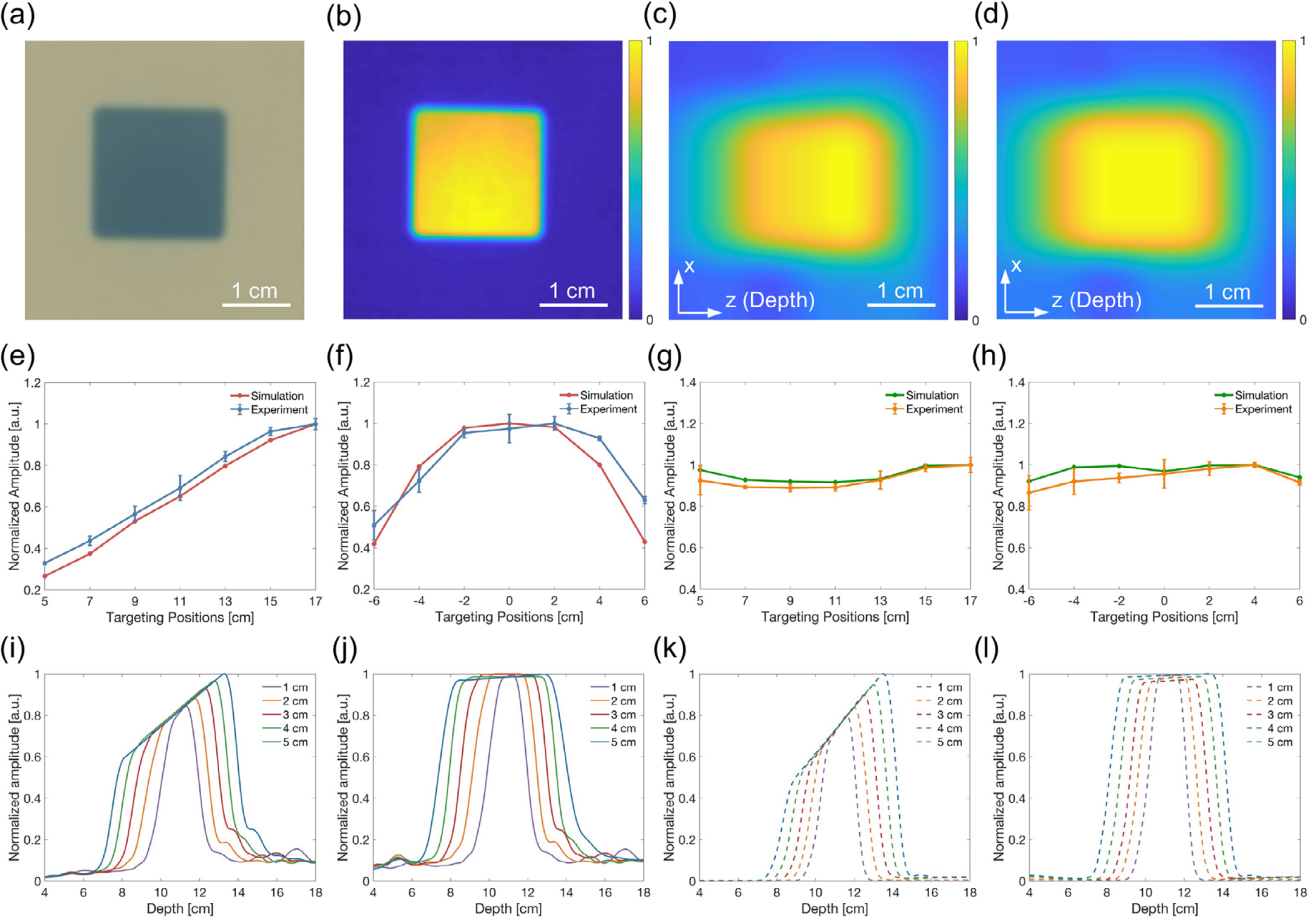
Quantification of radiation beams before and after compensation. (a) A 2 cm × 2 cm beam deposited on the radiochromic film. (b) Normalized pseudocolor image extracted from (a). (c) A 2D cross‐sectional image of dose distribution obtained from iRAI of the 2 cm × 2 cm beam. (d) Compensated image of (c) using the correction factors derived from experimental detection sensitivity. (e) Curves of normalized amplitudes as a function of the z‐direction, comparing experimental and simulated results. (f) Curves of normalized amplitudes measured at seven positions along the x‐direction, at z = 11 cm from the MAT surface. (g) and (h) Amplitude curves extracted from the compensated iRAI images, corresponding to (e) and (f), respectively. (i) Experimental depth‐dependent dose profiles for varying beam sizes. (j) Dose profiles derived from compensated iRAI images, corresponding to the experimental results in (i). (k) Simulated depth‐dependent dose profiles for varying beam sizes. (l) Corresponding profiles extracted from compensated simulated images shown in (k).

Figure 5a and 5b show the dose distribution from a 2 cm × 2 cm radiation beam captured by a radiochromic film and its corresponding normalized pseudo‐color image, serving as the ground‐truth dose distribution. Quantification was performed by comparing iRAI experimental data with K‐Wave MATLAB simulations. Figure 5c presents a 2D cross‐sectional image of the iRAI‐reconstructed dose distribution corresponding to the 2 cm × 2 cm beam shown in Figure 5b. Figure 5d shows the compensated image of Figure 5c, achieved by element‐wise multiplication with a correction factor volume derived from the experimentally measured detection sensitivity volume. As observed in Figure 5c, the pixel amplitude on the right side was higher than that on the left. After compensation, Figure 5d presents a more uniform amplitude distribution within the beam region. A further analysis of the compensation effectiveness is provided by averaged dose image intensities. Figure 5e and 5f show the normalized amplitudes of the pre‐compensation data across different axial and lateral positions, respectively, demonstrating close agreement between iRAI and simulation results. Before compensation, the axial amplitude increases steadily with distance, while the lateral amplitude is higher at the center and lower at the edges, indicating non‐uniform detection sensitivity. Specifically, Figure 5f exhibits an asymmetry in the experimental curve, with the left side showing lower amplitudes than the right. This difference likely results from the low sensitivity of several transducer elements on one side of the 2D MAT. Post‐compensation, as presented in Figure 5g and 5h, variations across locations decrease significantly in both experimental and simulation data, with all normalized amplitudes approaching 1.

Figure 5i presents the experimental dose profiles for beams of varying sizes (1–5 cm), all directed at a depth of 11 cm from the 2D MAT, showing distinct axial dose profiles. In comparison, Figure 5j shows the compensation results for the profiles in Figure 5i. Correspondingly, the simulated profiles before and after compensation are shown in Figure 5k and 5l, respectively. Sharp beam boundaries and rapid dose fall‐offs were observed across all beam sizes, indicating the accurate dose mapping capability of iRAI. The pre‐compensation profiles show an upward slope, indicating increasing amplitude with depth, which reflects enhanced detection sensitivity in the beam target regions. This result is consistent with the detection sensitivity map in Figure 4. After compensation, the profiles become more uniform, indicating that the method effectively reduces dose mapping errors caused by non‐uniform detection sensitivity.

### 3.3 C‐shape treatment plan quantification

**FIGURE 6:**
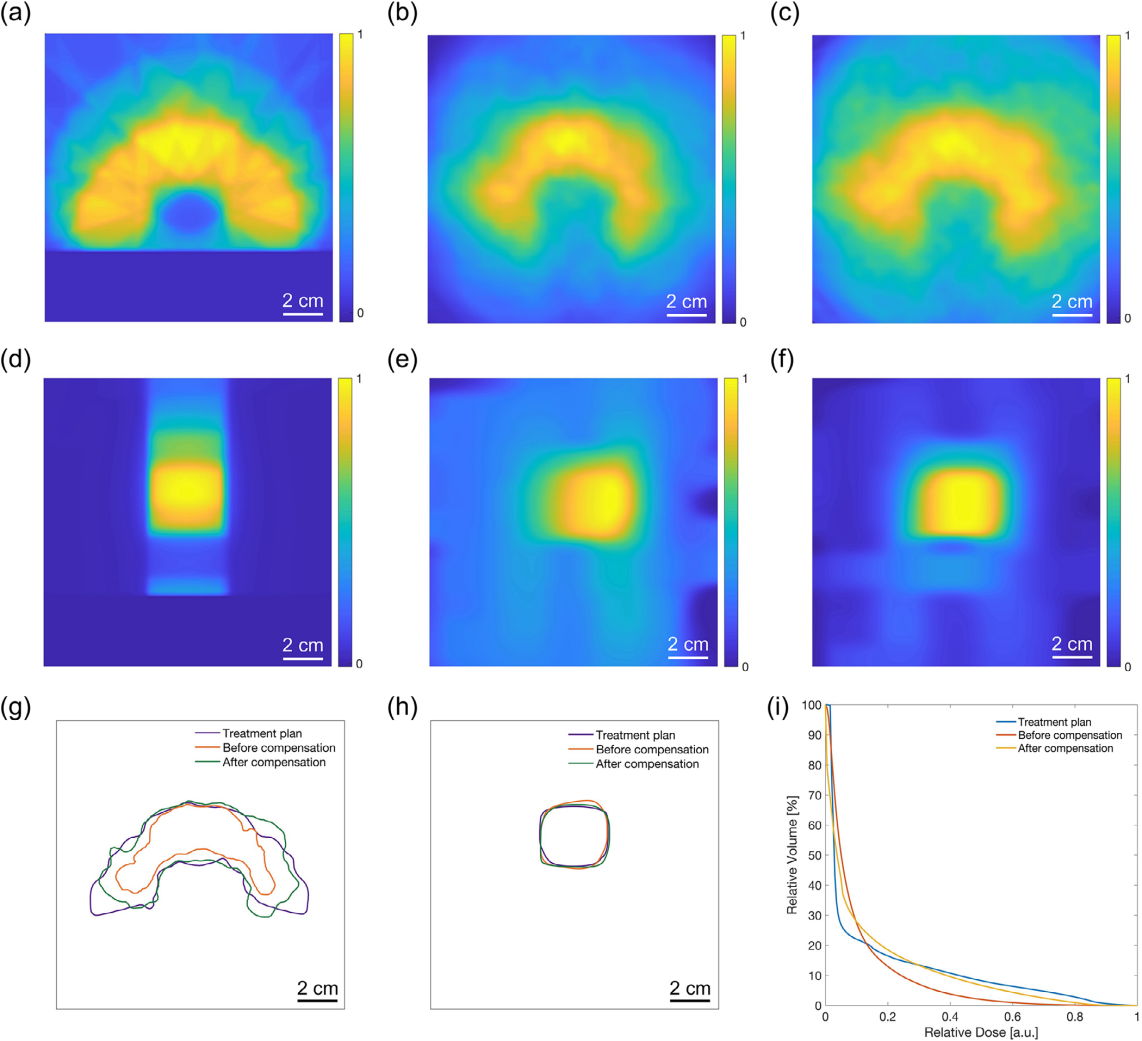
Reconstructed iRAI images and quantitative evaluation of the C‐shaped treatment plan. (a) Axial cross‐sectional image of the treatment plan at the mid‐plane of the C‐shape along the z‐axis. (b) Corresponding cross‐sectional image from the reconstructed iRAI image before compensation. (c) Axial cross‐sectional image after compensation. (d) Sagittal cross‐sectional image of the dose distribution from the treatment plan. (e) Corresponding sagittal cross‐sectional image from the reconstructed iRAI image before compensation. (f) Sagittal cross‐sectional image after compensation. The amplitude in all images was normalized. (g) Comparison of 80% isodose lines in axial cross‐sections from the treatment plan, pre‐compensation iRAI image, and post‐compensation iRAI image, corresponding to (a), (b), and (c). (h) Comparison of the 80% isodose lines in sagittal cross‐sections from the treatment plan, pre‐compensation iRAI image, and post‐compensation iRAI image, corresponding to (d), (e), and (f). (i) Comparison of DVHs based on volumetric dose distributions from the treatment plan, pre‐compensation, and post‐compensation iRAI images.

Figure 6a illustrates an axial (x‐y) cross‐section of the C‐shaped treatment plan within a selected 15 × 15 × 15 cm^3^ volume, showing the corresponding dose distribution. The raw iRAI data for this treatment were reconstructed into a volumetric image using the delay‐and‐sum algorithm in MATLAB, and the matching slice was extracted and presented in Figure 6b. The compensated slice, obtained after applying the correction factors, is displayed in Figure 6c. The amplitudes in all the three cross‐sectional images were normalized. Visually, the original treatment plan cross‐section shows a well‐defined C‐shape with a strong central region. In contrast, the pre‐compensation iRAI image shows reduced signal intensity and diffuse boundaries. After compensation, the iRAI image shows clear improvement, with corrected signal intensities and enhanced boundary definition. Figure 6d, 6e, and 6f present sagittal cross‐sections of the C‐shaped treatment plan, the pre‐compensation iRAI image, and the post‐compensation iRAI image, respectively. Overall, the dose distribution in all three images shows a roughly rectangular pattern. Before compensation, the signal appears lower on the left and higher on the right, primarily due to the non‐uniform detection sensitivity of the 2D MAT. After voxel‐wise compensation using the correction factors, the sagittal cross‐section shows a more uniform amplitude distribution.

The 80% isodose lines, recognized as a key clinical dosimetry benchmark,^29^ are presented in Figure 6g to compare high‐dose region alignment across the three axial cross‐sectional images. The isodose line of the original treatment plan presents a well‐defined C‐shape, while the pre‐compensation line shows an inward shift, indicating signal attenuation and shape distortion due to the non‐uniform sensitivity of the transducer. After compensation, the isodose line aligns more closely with the original treatment plan, especially with an expanded right wing that better conforms to the target geometry. However, some discrepancies remain along the outer boundaries, indicating that full restoration of the original structure has not been achieved. Similarly, Figure 6h presents the 80% isodose lines corresponding to the sagittal cross‐sections in Figure 6d‐6f. The treatment plan shows a well‐defined rectangular shape with smoothly rounded corners. Before compensation, the isodose line displays minor deviations, including a slight outward shift in the superior region and an inward displacement in the inferior region. After compensation, the isodose line demonstrates improved geometric conformity, closely aligning with the original treatment plan, especially along the superior and inferior edges. Although minor discrepancies remain, the correction led to better overall dose uniformity and spatial accuracy. Figure 6i shows dose‐volume histogram (DVH) curves derived from the 3D dose distributions of the treatment plan, the pre‐compensation iRAI image, and the post‐compensation iRAI image. The results indicate that before compensation, a larger volume received lower doses compared to the treatment plan, whereas after compensation, the dose distribution aligned more closely with the intended treatment. Quantitatively, the RMSE between the pre‐compensation DVH and the treatment plan DVH is 6.92, while the RMSE between the post‐compensation DVH and the treatment plan is reduced to 4.85. Furthermore, the 3D global gamma analysis with 5%/5 mm DD/DTA was calculated, increasing from 56.86% before compensation to 78.24% after compensation. Both the DVH and gamma pass rate results demonstrate that the accuracy of iRAI dose reconstruction improved after compensation.

## 4 DISCUSSION

This study demonstrates that compensating for the non‐uniform detection sensitivity of the 2D MAT can substantially improve dose mapping accuracy in iRAI. The detection sensitivity of the 2D MAT was characterized through both computational simulations and experimental measurements. These sensitivity maps were used to derive correction factors, which were subsequently applied to compensate for spatial sensitivity variations. In radiation beam quantification, compensation effectively corrected intensity asymmetry, leading to more uniform dose distributions. The compensated dose profiles became more uniform across depth, changing from sloped to flat in both experimental and simulated results, confirming the effectiveness of the correction method. For the C‐shaped treatment plan, compensation enhanced image quality by restoring signal strength and improving boundary definition. Quantitatively, the RMSE between the DVH derived from the iRAI volumetric image and that from the treatment plan was initially 6.92, and decreased to 4.85 after compensation, while the 3D global gamma pass rate with 5%/5 mm DD/DTA increased from 56.86% to 78.24% after compensation, demonstrating the effectiveness of the compensation method for improving iRAI dose mapping accuracy.

While this study demonstrates advancements in dose mapping using iRAI, some limitations must be acknowledged. First, the compensation method was applied in a homogeneous phantom, which does not represent the complexity of real tissue structures. Due to tissue and organ heterogeneity, variations in radiation energy absorption, scattering, acoustic attenuation, and acoustic impedance can distort the detected signals, thereby reducing the accuracy of dose compensation when using the correction factors derived in this study. To address this challenge, advanced inverse problem‐solving techniques will be explored, including model‐based reconstruction algorithms that account for spatially varying physical properties, deep learning‐based correction methods trained on heterogeneous tissue data, and the incorporation of anatomical priors from CT or MRI to guide compensation. These strategies can be built on methodologies previously established in quantitative photoacoustic imaging (QPAI),^30^, ^31^ and have the potential to improve dose reconstruction accuracy under clinically realistic conditions. Second, the current method is only implemented offline, since voxel‐wise sensitivity compensation requires considerable computational resources due to the need for point‐by‐point processing across the entire reconstructed volume. To enable real‐time application, future implementations may rely on GPU acceleration and precomputed correction maps with interpolation to reduce the computational burden of voxel‐wise compensation. Solving these issues will significantly advance the clinical application of iRAI.

In future work, we will investigate iRAI performance using a human abdominal phantom composed of tissue‐mimicking materials that represent various organs and anatomical structures. Relevant physical parameters, such as tissue density, sound speed, Grüneisen coefficient, and x‐ray absorption and attenuation coefficients, will be incorporated to enable a more comprehensive evaluation.

## 5 CONCLUSIONS

In conclusion, this study presents a novel, spatially correct, relative dosimetry iRAI approach for real‐time radiation dose measurement. By incorporating sensitivity compensation, the proposed method improves dose mapping accuracy. The results demonstrate that correcting for detection sensitivity variations significantly enhances the agreement between the iRAI images and the treatment plan, highlighting the potential of iRAI to improve precision in clinical RT. Future studies will focus on validating iRAI in more complex and realistic human models to further assess its clinical applicability.

## CONFLICT OF INTEREST STATEMENT

The following authors have previously disclosed a patent application (no. US12102843B2) that is relevant to this manuscript: P.L.C., I.E.N., W.Z. and X.W. Other authors have no conflicts to disclose.

## Data Availability

Data will be made available on request.
